# Complexome Profiling: Assembly and Remodeling of Protein Complexes

**DOI:** 10.3390/ijms22157809

**Published:** 2021-07-21

**Authors:** Ilka Wittig, Pedro Felipe Malacarne

**Affiliations:** Institute for Cardiovascular Physiology, Goethe University, 60590 Frankfurt, Germany; Malacarne@vrc.uni-frankfurt.de

**Keywords:** complexome profiling, mass spectrometry, protein complexes, protein–protein interaction, assembly, remodeling, data repositories

## Abstract

Many proteins have been found to operate in a complex with various biomolecules such as proteins, nucleic acids, carbohydrates, or lipids. Protein complexes can be transient, stable or dynamic and their association is controlled under variable cellular conditions. Complexome profiling is a recently developed mass spectrometry-based method that combines mild separation techniques, native gel electrophoresis, and density gradient centrifugation with quantitative mass spectrometry to generate inventories of protein assemblies within a cell or subcellular fraction. This review summarizes applications of complexome profiling with respect to assembly ranging from single subunits to large macromolecular complexes, as well as their stability, and remodeling in health and disease.

## 1. Introduction

The function of many proteins often requires stable or dynamic associations with other biomolecules, e.g., proteins, nucleic acids, carbohydrates or lipids in order to form large macromolecular assemblies. Protein complexes can be transient or stable and frequently need additional factors for the coordinated assembly of individual subunits into mature and functional macromolecular entities [1,2]. Conserved interaction sites allow for competitive docking of different proteins. This enables proteins to bind to various interaction partners at the same binding site forming unique complexes with differing functions [3]. Alteration of cellular conditions induced by stress or availability of substrates (e.g., nutrients and oxygen) requires dynamics of protein interactions [3,4]. Therefore, the formation and remodeling of protein complexes need to be controlled. Altered stability and dynamics of protein complexes is often associated with disease development and progression [1,5]. Studies on protein function or malfunction in a disease state employ interaction proteomics to identify components involved in the molecular mode of action and to gain deeper insight into pathomechanisms [1]. Targeted interaction proteomics may involve affinity enrichment protocols that rely on antibodies [6,7], affinity tags [8,9,10], or in cell biotin labeling [11] coupled to quantitative mass spectrometry. The advantage of using targeted strategies is an enrichment of the protein assembly, which enables in depth characterization of its interacting components. The use of proximity-dependent labeling, e.g., biotin ligase fusion proteins (BioID), facilitates identification of transient protein interaction events in vivo [12]. High throughput pulldown strategies discover protein–protein interactions and can be even adapted to the enrichment of protein assemblies with nucleic acids and lipids [13,14,15]. Although pull down approaches are powerful, widely used, and able to identify even scarce interaction partners, they are limited to availability of antibodies or the possibility to use enrichment tags. Furthermore, as enrichment of one protein of interest pulls down a mixture of different complexes along with itself, it is not possible to distinguish between individual complexes formed by the same protein, e.g., assembly intermediates, different states of complex remodeling, and the impact on additional macromolecular complexes. 

**Figure 1:**
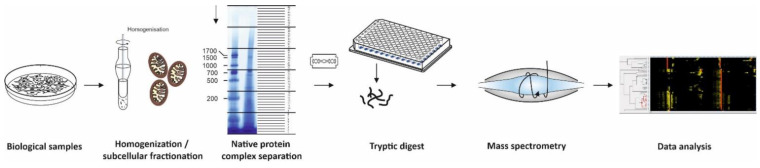
Workflow of complexome profiling. Sample preparation of biological samples include homogenization, subcellular fractionation, mild solubilization, and separation of native protein complexes by BNE or density gradients. Even fractions are placed into microtiter plates and digested with trypsin. Peptides are analyzed by quantitative mass spectrometry using LC- MSMS to gain information on peptide sequence. Data analyses comprise peptide and protein identification and quantification, hierarchical clustering of proteins with similar migration in the gels or gradients. These abundance profiles contain comprehensive information on protein complexes, subcomplexes, and super-assemblies.

Complexome profiling (CP) can overcome these limitations as this untargeted strategy collects information of the entire interactome within a biological sample without enrichment by specific antibodies or tags [16,17,18]. Biochemical fractionation from density gradient centrifugation or native electrophoresis followed by quantitative mass spectrometry are used to generate protein interaction maps of native protein complexes with additional information on their native mass, stoichiometry, and recently protein turnover within protein complexes (Figure 1) [16,19]. Comparison of interaction profiles from a series of samples in one experiment gathers valuable insights into dynamic processes of protein complexes. Very popular in studies of the oxidative phosphorylation system (OXPHOS) complexes in mitochondrial disease, CP was further developed to investigate stable RNA-protein complexes [20]. This review provides a concise survey on the complexome profiling method and applications to elucidate composition and dynamics of macromolecular complexes. Additional combinations with stable isotope labeling of amino acids in cell culture (SILAC) [21] and tandem mass tag labeling (TMT) [22] recently expanded the spectrum of applications. As comprehensive CP data sets contain more information than addressed in the initial publication, complexome profiles are rich in untapped accessible data uploaded to repositories for additional investigations by the scientific community. Several intuitive bioinformatics tools have become available in recent years, proving themselves to be useful to analyze interaction networks leading to further insights into the molecular characteristics of cell function. 

## 2. Workflows to Study Composition, Dynamics, and Remodeling of Protein Complexes

Complexome profiling [17,18], protein correlation profiling (PCP) [23], and co-fractionation mass spectrometry (CoFrac-MS) [3] follow essentially the same strategy and workflow: Biological samples (e.g., cells, isolated organelles, tissue specimens) are homogenized and solubilized under mild conditions to maintain native complexes led by separation in native gels or density gradients (Figure 1). Upon enzymatic digestion of each fraction, peptides are subsequently analyzed by quantitative mass spectrometry. Identified proteins with similar appearance within the biochemical fractions are hierarchically clustered and considered as candidates to form a protein complex (Figure 1). 

In general, any biochemical native separation technique in combination with mass spectrometry is able to generate comprehensive protein-interaction maps. The initial strategy of protein correlation profiling (PCP) used sucrose density gradient fractionation to identify new components of human centrosomes [23]. Density gradient centrifugation is the appropriate choice whenever separation of very large cellular components, e.g., cellular organelles, ribosomes, large oligomeric states of protein complexes, lipid rafts, and microsomal fractions, are required. Protein profiles with a similar appearance within the fractions are hierarchically clustered to generate an unbiased interaction survey or sorted by available information on their sub-organelle affiliation [23,24,25,26,27,28]. 

Although very useful for studies on large assemblies of biomolecules, density gradients have inherent low resolution, require a large amount of sample, and it is difficult to differentiate between co-purification in a fraction or an actual physical protein–protein interaction. 

Depending on the separation resin, size exclusion chromatography (SEC) isolates protein complexes up to several MDa and is suitable to analyze a broad range of cellular protein complexes [29,30,31]. In addition, SEC has the advantage of directly coupling protein complex separation with native electrospray ionization mass spectrometry [32]. When working with scarce samples, e.g., patient biopsies or primary cell culture, the sample amount is limited and not enough for the use of density gradients and SEC based CP [30,33]. Blue native electrophoresis (BNE) overcomes this limitation. 

BNE became a very robust and reproducible, micro-scale high resolution separation method to examine composition of protein complexes in a broad range of samples from bacterial membranes, soluble subcellular components, and membrane fractions from eukaryotic cells to tissue specimens from patients [34]. Standard BN gels cover a mass range from several kDa to 10 MDa. Whenever large protein complexes and mega-assemblies are in focus, a special large pore gel enables separation of up to 60 MDa [35]. Lanes of BNE gels are fixed, stained with Coomassie dye, divided into even slices, and digested with trypsin (Figure 1). The resolution of complexome profiles increases with the number of slices and differs between published approaches from manually cut 24, [18], 48 [36], and 60 [17] gel pieces up to several hundreds of sub-millimeter slices from BN-gels by a cryo-microtome [37,38]. Using BN gels for CP the complexity of each fraction in the high molecular mass region is with a few hundred protein identifications considerably lower compared to the region of individual proteins closed to the electrophoretic front. For this reason, a short effective gradient of 30–45 min in liquid chromatography-mass spectrometry (LC-MSMS) runs is suitable to identify the majority of proteins in each fraction. Since a few years ago, we divide all our BNE lanes into 48 slices and analyze the resulting peptides in short gradient LC-MSMS runs for approximately one hour each. A standard complexome analysis of a mitochondrial preparation to investigate assembly defects of a patient takes approximately 48 h per sample. Thus, the analysis, including control and a second patient, can be completed within a timeline of one week [36]. 

The aim of each complexome profiling experiment is to gain information of native protein complexes and to compare the appearance and abundance of assemblies under different conditions or in disease states. Biological samples for CP range from bacteria [39,40], yeast [41] or cell culture [4,17,18,19,21,36,42,43,44,45,46,47,48,49,50,51], tissue or organ specimens from patients and animals [17,19,37,38,52,53] to plants [54,55,56,57,58,59,60,61,62]. Biochemical preparations of subcellular fractions (e.g., mitochondria, microsomes) reduce sample complexity for a more in depth complexome analysis [17,51]. Lysis conditions with low ion strength in solubilization buffers and mild detergents keep protein complexes in a near native state. Solubilization protocols including suitable neutral detergents have been described for various kinds of samples [34,63]. 

In recent years, further developments on CP (Table 1) included steps in the sample preparation discussed in the following sub-sections; (1) to enable complex assembly to be monitored, (2) to improve the sample comparison using SILAC and TMT, to monitor protein complex, (3) remodeling, (4) turnover and repair, (5) to gain structural information on complex conformations by applying protein crosslinkers, and (6) to identify RNA-protein complexes.

### 2.1. Assembly and Stability of OXPHOS Complexes

Assembly of protein complexes [70] or even further association of several protein complexes require a coordinated process with the help of chaperons also called assembly factors. In recent years, classical CP became a very popular tool to study the significance of individual subunits and assembly factors of the OXPHOS complexes in bacteria [39,40,61] and mitochondria from mammals [17,21,22,27,28,36,41,42,43,44,45,46,47,48,49,50,53,64,65,66] and plants [54,55,56,57,60,62]. 

TMEM126B was the first assembly factor identified by complexome profiling [17]. Rat heart mitochondria were solubilized with the mild detergent digitonin and separated by BNE [34] and also by large-pore BNE [35]. Upon hierarchical clustering an association of ACAD9, NDUFAF1, ECSIT with one protein of unknown function TMEM126B drew our attention. Knock down experiments and functional analysis confirmed TMEM126B as an essential factor for complex I assembly in a complex with the other proteins forming the mitochondrial complex I assembly factor complex (MCIA). A few years later, patients with mutations in the TMEM126B gene were identified and assembly defects were characterized by CP [46,50]. Another approach used CP to elucidate essentially the complete step-wise assembly sequence of mitochondrial respiratory chain complex I in human mitochondria [42]. Upon inhibition of mitochondrial translation by chloramphenicol treatment for several days, mitochondrial respiratory chain complexes that contain mitochondrial encoded subunits appeared disassembled. After drug removal a stepwise assembly of complex I was monitored by recording complexomes of several time points. With increasing time, it was possible to follow the formation of early building blocks to association of central modules to final assembly stages until the fully matured complex I and the respiratory supercomplexes containing complex I, III and IV. Five different subassemblies could be followed with known assembly factors to complete a matured complex I [42]. 

In recent years, CP has become a standard tool to study assembly defects in patients with mitochondrial disorders. Profiles from patients mainly show typical accumulation of assembly intermediates that can be used for the interpretation of the significance of a subunit, an assembly factor in the assembly pathway or a ribosomal protein [36,44,45,46,47,48,49,50,64,71]. In contrast to the de novo synthesis of OXPHOS-complexes upon treatment with a translation inhibitor [42], patients with defects in the NDUFA6 [45], NDUFC2 [36], and COX4I1 [43] showed, under steady state levels, a clear association of stalled assembly intermediates with other respiratory chain complexes, suggesting that completion of individual complexes is not a prerequisite for supercomplex formation [48].

### 2.2. Multiplexing CP

Most initial CP approaches used label free quantification. As all fragments from each gel lane or density gradients have to be measured in single MS runs, such approaches need extensive machine time and the data from several separation gradients have to be merged. The introduction of metabolic labels in SILAC-based CP allows direct comparison of protein migration and the abundance of two or three different samples in one native gel lane [21,65]. Although this duplex approach introduces more complexity in data analysis, the advantage is a precise annotation of the differences in the complexes between two different conditions. This strategy determined the co-existence of structurally distinct respirasomes in human cells [21]. Quantitative Density Gradient analysis by Mass Spectrometry (qDGMS) combines SILAC with the separation of protein complexes in a density gradient followed by quantitative mass spectrometry. This approach was applied to study human mitochondrial ribosomes [26]. The advantage in contrast to label free quantification is a direct comparison of two samples in one gradient. Technical bias during sample preparation making it difficult to identify biological variations can be excluded [26,28].

SILAC-based CP is limited to samples that can be metabolically labeled either in cell culture or with appropriate isotope containing diet [72]. In addition to metabolic labeling in cell culture, chemical labeling during sample preparation with tandem mass tags (TMT) enables multiplexing of up to 16 samples in one CP. This strategy using reporter ions for quantification was effective in recording the assembly of respiratory chain complexes after removal of mitochondrial translation inhibitor chloramphenicol in a time dependent manner [22]. Less reporter ion variation in complexes unaffected by chloramphenicol treatment illustrated the power of using tandem mass tags in multiplexed CP for research and diagnostics [22]. 

### 2.3. Remodeling

Shifts in availability of substrates, oxygen, and various stresses require cellular adaptation to new conditions. This includes a fast response mechanism on the level of proteins and complexes such as in signaling pathways and metabolic enzymes or a long term response on the level of gene expression. CP was recently used to study the molecular consequences on respiratory complex I during chronic hypoxia in the human leukemia monocytic cell line THP-1 [4]. This study explored an HIF1-α dependent complex I assembly defect in response to the degradation of the assembly factor TMEM126B [4]. Another impressive example of remodeling the whole respiratory chain identified by CP was reported in plant mitochondria of the European mistletoe (*Viscum album*) [62]. This obligate semi-parasite living on branches of trees lacks complex I and exhibits remarkably stable supercomplexes containing complex III and IV. A differential CP approach on plant leaf mitochondria identified dynamics of protein complexes in the presence and absence of light to integrate biochemical processes during day and night [54].

### 2.4. Turnover of Subunits within Protein Complexes

Protein complex assembly has been frequently studied in cell culture from fast dividing cell lines or patient fibroblasts [5,73]. All these studies focus on de novo complex assembly from single subunits to mature complexes. The situation in postmitotic tissues might in contrast rather reflect an equilibrium stage of turnover with a balance of biosynthesis and degradation. It is an important question whether a protein complex is built from scratch or whether there are cellular mechanisms to service protein complexes to maintain function. 

**Figure 2:**
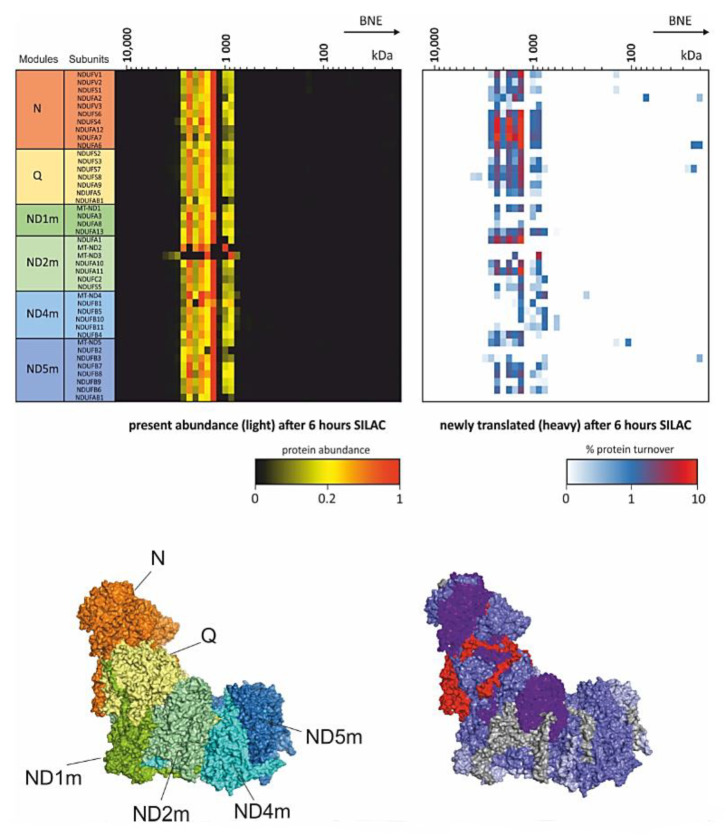
pSILAC Complexome profiling. Mouse myocytes were differentiated into myotubes and incubated for 6 h with SILAC medium. Heatmaps (**upper panel**) show the present light labeled complex I subunits (**left**) and the portion of exchanged newly translated heavy subunits (**right**). **Lower panels** indicate the modules of complex I (**left**) according to Formosa et al. [73] and the turnover of subunits (**right**) within the mouse complex I structure [66,76]. These complexome profiling data originally used in [19] were reanalyzed from PRIDE PRoteomics IDEntifications (PRIDE) archive database identifier PXD017465 [19,77].

Introducing SILAC as a pulse [74] for several hours followed by CP workflow gives insights into dynamics within protein complexes. This strategy enables the study of remodeling and repair in protein complexes [19,66]. Of mention in that respect is an experiment carried out in differentiated mouse myotubes from C2C12 cells (Figure 2). These myotubes were pulsed for 6 h with SILAC and the turnover of single subunits within the respiratory chain complex I was studied [19]. This experiment showed that parts of the peripheral arm of complex I was replaced. Although pulse SILAC (pSILAC) experiments analyzing total cell lysates detected a general fast turnover of subunits of the N- and Q- module of complex I [75], here it was clearly shown, that replacement takes place within a protein complex. In the same study, the matrix protease CLPP could be identified as an important component in this complex I maintenance pathway. Cells from the CLPP knockout mouse were investigated with the same setting of pSILAC-CP. In contrast to the control, the N-module of complex I showed very low turnover and newly synthesized intermediates of N-module subunits accumulated as an intermediate [19]. Other subunits of the Q module NDUFA6 and NDUFA7 showed comparable level of subunit turnover suggesting that CLPP is essential for the service of the N-module in assembled respiratory supercomplexes. 

Another component functioning as a service factor is DNAJC30. Patients with defects in DNAJC30 develop recessive Leber’s hereditary optic neuropathy [66]. Complexome profiles from these patients showed comparable abundance of fully assembled supercomplexes but had low complex I activity. As no assembly defect was detected, and DNAJC30 was identified to bind to complex I subunits in pull down experiments and complexomes, the turnover rates of OXPHOS complexes were monitored in several patients. Significant lower turnover was detected in the direct interaction partner subunits NDUFA6 and NDUFA7 of DNAJC30. Indeed, these subunits exhibit the highest turnover in wildtype complex I and located at the peripheral arm spanning the N- and Q-module (Figure 2). It was concluded that NDUFA6 and NDUFA7 need to be removed before any maintenance can take place on complex I. This suggests that DNAJC30 is upstream of CLPP in the service plan of complex I [66].

### 2.5. Structural Information of Protein Complex Conformation

Chemical crosslinking MS (XL-MS) enabled structural investigations on various protein complexes. Crosslinkers used in MS are mainly homo-bifunctional reagents with two reactive sites to covalently bridge two lysine residues in a close neighborhood to capture a dynamic interaction and conformation [78]. Various cleavable and non-cleavable spacer arms with different lengths extended the options to study protein complex conformations [33]. In-solution crosslinking prior to native separation in CP is a challenge. The resolution of protein complexes in the native gel decreases with the amount of introduced cross-linker and results in difficult data interpretation. To use interaction-specific cross-linkers in CP and to gain information on molecular interaction across protein assemblies the workflow was recently adapted for in-gel cross-linking MS (IGX-MS) [79]. Cross-linking in-gel has turned out to be easier to control compared to in-solution and emerged as a powerful strategy that allows compositional and interaction specific distance measurements to be used for further refining structural models [79]. As a proof of principle, IGX-MS was applied to measure the state-specific crosslinking of isolated complex I and ATP synthase from bovine heart mitochondria in a few BN-gel bands. Theoretically, such an approach is scalable and can give important structural information to all isolated protein–protein interactions in a BN-gel or other separating techniques [80]. However, it will be still limited to a subset of protein complexes and focused interests as analysis tools for comprehensive crosslinking studies in CP are not yet available. 

### 2.6. RNA-Protein Complexes

Most of the CP applications are protein-only approaches. Introduced RNA sequencing (RNA-seq) in a complexome analysis expand the technique [20]. Gradient profiling by sequencing and mass spectrometry (Grad-Seq) is a hybrid complexome analysis that combines density gradient centrifugation with quantitative protein mass spectrometry with RNA-seq and has the power to quantitatively profile transcripts or non-coding RNA that co-segregate with proteins. [20,58,67,68,69].

## 3. Data Analysis

After mass spectrometry, all slices are analyzed with standard proteomics software tools such as: MaxQuant, Proteome discoverer, PEAKS [81,82,83]. In this line the publicly available MaxQuant software has been used frequently and despite other proteomics approaches (e.g., pull down analysis, complete proteomes) that use biochemical comparable fractions and single peptide quantification, many publications on CP use a quantification value that is independent of the comparable faction protein residents. One prominent value is the intensity based absolute quantification value (IBAQ) when using MaxQuant [84]. Initially used to spike a standard to calculate absolute protein amounts, IBAQ also serves as an important quantification value that correlates with the absolute protein abundance [85]. As fractions in CP comprise biochemically different fractions, all quantification values that compare single peptides and use best peptide value (e.g., the best 3 like in PEAKS), are not well-fit strategies for BN gel fractions. IBAQ values represent the sum of all peptide intensities divided by a theoretical number of tryptic peptides of the protein [84]. If a small protein gives only a few peptides, the divider is also small and theoretically brings subunits with the same stoichiometry to one level. Inspecting available complexomes and comparing the IBAQ values, e.g., complex I subunits, however showed that this approach is not suitable for all proteins. For example, membrane spanning domains have a general lower amount of identified peptides and also proteins which are difficult to digest are underrepresented. Nevertheless, IBAQ values can be used to explore portions of bound proteins, e.g., if an extra factor like the service factor DNAJC30 binds to a subset of complex I. For SILAC and pSILAC-CP it is very useful that MaxQuant displays IBAQ values as light and heavy and enables comparison and calculation of protein turnover rates [19,66]. 

Once a protein list is created, quantification values of each protein within the fractions are used to compare migration behaviors in native gels or sucrose density gradients. That can be accomblished by using several tools to analyze complexome profiling data (Table 2). Co-separated complexes with known stoichiometry serve as internal standards and are used for native mass calibration. The software tool NOVA was developed to analyze data from complexome profiles. NOVA is an intuitive tool and implements several hierarchic clustering algorithms, different distance measures (e.g., Euclidean distance, Pearson distance), and various normalization techniques together with options to generate 2D plots, heatmaps, and search functions [86]. Other proteomics data analysis tools e.g., Perseus and proteome discoverer have also been used to analyze complexome profiling data [17,87].

Furthermore, ComPrAn stands out as an additional tool to analyze complexomes and was initially developed for qDGMS data [28]. This freely available R-package provides analysis on peptide-level data, normalization, and clustering tools for protein-level data, include functions to compare changes of protein complex composition between two SILAC labeled samples and produce publication-ready figures. Another software to analyze CP is ComplexFinder. The python-based computational pipe line implements machine learning to better identify protein complexes whenever multiple complexes with varying protein composition escape identification by hierarchical clustering [90]. 

If profiles from serveral sets of experiments need to be compared, the COmplexome Profiling ALignment (COPAL) tool can be used to merge several profiles from different gel runs. Using COPAL, it was possible to detect remodeling of mitochondrial complexes in Barth syndrome [88].

Recently, the complexome profiling Data Resource (CEDAR) repository was installed. CEDAR includes a storage and information sharing platform to support the reuse of complexome profiling data [89]. Many researchers also uploaded their mass spectrometry raw data into the Proteomics Identification database (PRIDE) together with analysis data for reuse by the scientific community [77]. Most of the CP data contain much more information than used in the initial publication. Available data in these repositories serve as a gold mine to discover new protein interactions and to build networks in systems biology investigation. 

## 4. Conclusions

CP has become a very useful tool to study protein complexes, to investigate dynamic processes of complex assembly, for remodeling, and protein turnover. The implementation of SILAC and TMT for multiplexing CP enhances speed of analysis and comparison of multiple samples. Data from in-gel crosslinking MS will give important insights into the molecular dynamics of protein complexes and conformations in future. Pulse SILAC and closer studies on posttranslational modifications generate “profiles within profiles” to better understand protein complex remodeling and maintenance in intact cellular physiology. CP has been already used to understand the assembly defects in mitochondrial disorders and will be very helpful to explore cellular protein networks and the impact of protein complexes on disease development, progression, and the benefit of treatments. 

## Figures and Tables

**Table 1:** Versions of CP methods to examine inventory of protein complexes.

CP Methods	Description	References
Classic CP	Combines BNE, density gradient centrifugation or size exclusion chromatography with quantitative MS to generate interaction profiles	[4,17,18,19,36,37,38,39,40,41,42,43,44,45,46,47,48,49,50,51,52,53,54,55,56,57,59,60,61,62,63,64]
qDGMS	SILAC based comparison of protein complex profiles separated by a density gradient	[26,28]
High-resolution CP	Classical CP with sub-millimeter sampling of native gels to enhance resolution and data depth	[37,38]
SILAC-based CP	Processes two samples in one BN-gel for comparison	[21,65]
Multiplexed CP	Introduction of tandem mass tags (TMT) allows processing of multiple samples	[22]
pSILAC CP	Uses SILAC as a pulse to investigate protein turnover in complexes	[19,66]
Grad-Seq	Combines density gradient centrifugation, quantitative MS, and RNA-seq for the analysis of RNA-protein complexes	[20,58,67,68,69]

**Table 2:** Tools to analyze complexome profiling data.

Software/Database	Description	References
NOVA	Implements cluster analysis, visualization, native mass calibration and comparison.	[16,86]
COPAL	COmplexome Profiling ALignment (COPAL) aligns lanes for comparison of multiple samples	[88]
ComPrAn	R package to study protein assemblies	[28]
CEDAR	Online resource of CP data	[89]
ComplexFinder	Machine-learning based prediction of novel protein complexes	[90]

## Data Availability

Not applicable.
